# How to characterize figures of merit of two-dimensional photodetectors

**DOI:** 10.1038/s41467-023-37635-1

**Published:** 2023-04-19

**Authors:** Fang Wang, Tao Zhang, Runzhang Xie, Zhen Wang, Weida Hu

**Affiliations:** 1 State Key Laboratory of Infrared Physics, Shanghai Institute of Technical Physics, Chinese Academy of Sciences, Shanghai, 200083 China; 2 University of Chinese Academy of Sciences, Beijing, 100049 China

**Keywords:** Photonic devices, Optoelectronic devices and components

## Abstract

Photodetectors based on two-dimensional (2D) materials have been the focus of intensive research and development over the past decade. However, a gap has long persisted between fundamental research and mature applications. One of the main reasons behind this gap has been the lack of a practical and unified approach for the characterization of their figures of merit, which should be compatible with the traditional performance evaluation system of photodetectors. This is essential to determine the degree of compatibility of laboratory prototypes with industrial technologies. Here we propose general guidelines for the characterization of the figures of merit of 2D photodetectors and analyze common situations when the specific detectivity, responsivity, dark current, and speed can be misestimated. Our guidelines should help improve the standardization and industrial compatibility of 2D photodetectors.

## Abstract (web summary)

The lack of a standardized approach for the characterization of the performance of 2D photodetectors represents an important obstacle towards their industrialization. Here, the authors propose practical guidelines to characterize their figures of merit and analyse common situations where their performance can be misestimated.

## Introduction

The combination of two-dimensional (2D) semiconducting, insulating, and metallic materials with remarkable optoelectronic properties offers promising solutions for the development of next-generation photodetectors^1–3^. However, the performance characterization of 2D photodetectors lacks standardization, which makes it difficult for many reports to offer an objective and unified performance comparison. On the one hand, performance overestimation is often attributed to the fact that the optical effective area of 2D materials is difficult to determine compared to traditional materials. On the other hand, when the photocurrent of the device is measured via laser sources, the estimation of the Gaussian beam diameter based on the outline of the laser spot also causes errors in calculating the power density of the device. Furthermore, the noise evaluation with dark current is controversial and the definition of response time parameters can be equivocal. Indeed, many reports of response times are based on non-complete square wave periods and cannot realistically present the speed of photodetectors.

Undefined and non-standard characterization methods have seriously hindered the development of 2D photodetectors, making it impossible to establish valid comparisons of photodetector performance between different materials and architectures^4^. Here, we discuss the performance characterization of 2D photodetectors systematically, including the role of the device active area and the power density of incident light, the measurement protocol used for the photodetector dark current and noise, and how these definitions impact the relevant noise equivalent power, responsivity, and specific detectivity. Also, the time parameters of response time and response bandwidth are discussed. We introduced common situations when important parameters can be easily misestimated and the resulting influence on the extraction of specific detectivity and speed. Finally, we illustrate the impact of the photodetection mechanism on the performance of 2D photodetectors.

## Characterization and performance evaluation of 2D photodetectors

More and more 2D photodetectors have been reported with significant values of specific detectivity (D*) and response bandwidth *f*_*c*_. Table 1 summarizes the performance parameters related to D* and *f*_*c*_. Such scenarios include misestimation of the optical effective area *A*_*d*_ and noise ${i}_{n}$, misestimation of operating frequency *f* and so on. Firstly, with the participation of mechanisms such as nano-optical effects, the actual response area of the device at the near-wavelength scale is often higher than the area estimated from the device photomicrograph. In addition, the drift of electron-hole pairs caused by the external electric field will also affect the collection range of photogenerated carriers in the device, and then affect the optical effective area. Moreover, the noise ${i}_{n}$ can be easily underestimated by using formula ${i}_{n}=\sqrt{2{qI}\triangle f}$ or ${i}_{n}=\sqrt{2{qI}\triangle f+\frac{4{k}_{B}T}{{R}_{d}}\triangle f}$, where ${i}_{n}$ is the noise current, *q* is the elementary charge, *I* is the average current (including dark current and background radiation current), *k*_*B*_ is the Boltzmann constant, *T* is the measurement temperature, ${R}_{d}$ is the resistance, and $\triangle f$ is the operating bandwidth. This estimation only considers the white noise characteristic dominated at high frequency and ignores the frequency-dependent colored noise. Especially for 2D photoconductive photodetectors with generation-recombination (g-r) noise proportional to photoconductive gain ($G=\tau /{\tau }_{t}$, where $\tau$ is the carrier lifetime, ${\tau }_{t}$ is the carrier transit time), ignoring the gain-dependent noise component will lead to a significant overestimation of D*. In addition, another possibility for the misestimation of D* is neglecting the consistency of specific frequencies for the noise and response spectrum. In addition to the specific detectivity, noncanonical response time measurement can also lead to incorrect evaluation of response bandwidth *f*_*c*_. Standardized measurement and evaluation are essential for the sustainable development of 2D photodetectors. It could bridge the gap between the technology assessed in academic laboratories and industrial applications.

**Table 1:** Figures of merit of 2D photodetectors and sources of error in their estimation

${D}^{*}=\frac{\sqrt{A_d\triangle f}}{{NEP}}=R\frac{\sqrt{A_d\triangle f}}{{i}_{n}}=$ $\frac{{I}_{s}}{p{i}_{n}}\sqrt{\frac{\triangle f}{A_d}}$					${f}_{c}$
$A_d$	${i}_{n}$	$\triangle f$	$R=\frac{{I}_{s}}{{P}_{\lambda }}=$ $\frac{{I}_{s}}{pA_d}$		
			$p$	${I}_{s}$	
Easy to be underestimated when the bias voltage is applied or the near field effect is strong	Easy to be underestimated with ${i}_{n}=\sqrt{2{qI}\triangle f+\frac{4kBT}{{R}_{d}}\triangle f}$	Responsivity and noise should be measured at the same frequency, or it will result in incorrect estimation of ${D}^{*}$	Easy to be misestimated with $p=E/A_d$, where *E* is the laser intensity	*I*_*s*_ is proportional to *G* for photoconductive photodetectors	Easy to be overestimated with response time of non-complete cycle
	g-r noise is proportional to *G* when ${4\pi }^{2}{{f}^{2}\tau }^{2}\ll 1$			Gain bandwidth product *GBP* ∝ (*τ*/*τ*_*T*_)*τ*^−1^ $=$ ${\tau }_{T}^{-1}$	

D* is the specific detectivity, ${I}_{s}={I}_{l}-{I}_{d}$ is the net photocurrent, ${P}_{\lambda }$ is the incident light power, $p$ is the power density of incident light, ${i}_{n}$ is the noise current, *k*_*B*_ is the Boltzmann constant, *T* is the measurement temperature, ${R}_{d}$ is the resistance, $f$ is the frequency, $\triangle f$ is the operating bandwidth, and $A_d$ is the device effective area of 2D photodetector. ${I}_{l}$ and ${I}_{d}$ are measured light and dark current, respectively. For responsivity of device characterized in V/W, the noise current ${i}_{n}$ should be replaced with the noise voltage ${v}_{n}$. $R={{I}_{s}}/{{P}_{\lambda }}$ is the responsivity with unit of A/W. ${NEP}={{i}_{n}}/{R}$ is the noise equivalent power. For the case the voltage signal is measured, the responsivity is ${R}_{v}={{v}_{s}}/{{P}_{\lambda }}$ with unit of V/W. ${f}_{c}$ is the −3 dB bandwidth, and *t* is the response time. g-r noise is the generation-recombination noise. *GBP* is the gain bandwidth product, *τ* is the carrier lifetime, and ${\tau }_{T}$ is the carrier transit time. $\,G={\tau }/{{\tau }_{t}}$ is the photoconductive gain.

Furthermore, in Box 1 we discuss the impact of different photodetection mechanisms on the device performance in terms of device structure, *I–V* curves, scanning photocurrent mapping (SPM). Up to now, the parameters of most 2D materials photodetectors are obtained by laser measurements and overestimated. It is very necessary to distinguish different types of 2D photodetectors by physical mechanisms to further characterize figures of merit of 2D photodetectors.

Box 1 Impact of the physical mechanism enabling photodetection on the performance of 2D photodetectors The photoconductive effect is based on a single and homogeneous semiconductor. The corresponding device is usually comprised of a semiconductor and the same metal contact electrodes (panel a). Under light illumination, the photogenerated excess carriers need to be driven under the externally applied voltage and collected by the metal electrodes (panel b). Scanning photocurrent microscope (SPM) characterization measurements show that opposite photocurrents are detected nearby the metal-semiconductor interfaces under zero applied voltage (panel c). Furthermore, the photoconductive effect is closely related to the bandgap of semiconductors. The device configuration of the photovoltaic effect usually consists of a pn junction (panel d). The photogenerated electron-hole pairs are separated by the built-in electric field of the pn junction. The dark *I–V* curve is exponential. Under light illumination, non-zero open-circuit voltage and short-circuit current appear (panel e). The current mainly distributes among the pn junction region (panel f). The photoresponse wavelength is defined by the bandgap of p-type or n-type semiconductors. As the spot of the focused illuminated visible light is smaller than the size of the device channel, this light could possibly lead to a temperature gradient at the channel. This temperature difference between the different parts of the channel or the 2D materials-to-metal interface gives rise to the thermoelectric current and voltage (panel g). Aside from the focused light illumination, the temperature gradient is produced when the light absorption of different parts of the channel varies. Both dark and illuminated *I–V* curves for the photothermoelectric effect are linear (panel h). SPM measurements indicate that opposite photocurrent is generated in the entire channel and changes from the positive to the negative (panel i). The photoresponse does not depend on the bandgap of the materials. The bolometric effect consists in the increase or decrease of the resistivity of a temperature-sensitive material illuminated by light (panel j). The photocurrent is only observed by applying an external bias and does not rely on the wavelength of the incident light (panel k).
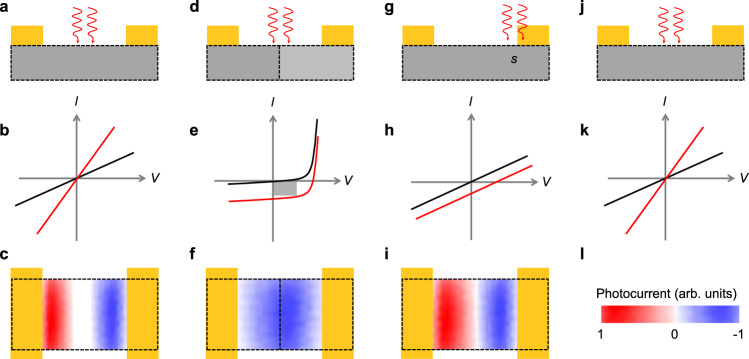

**a**–**c** Photoconductive effect, and corresponding *I*–*V* characteristic curve and SPM scanning result. **d**–**f** Photovoltaic effect, and corresponding *I*–*V* characteristic curve and SPM scanning result. **g**–**i** Photothermoelectric effect, and corresponding *I*–*V* characteristic curve and SPM scanning result. SPM and s represent scanning photocurrent microscope and semiconductor, respectively. **j**, **k** Bolometric effect, and corresponding *I*–*V* characteristic curve. **l** Scale bar of SPM results.

## Device effective area

The device effective area is an important parameter to effectively model the device and then obtain the various figures of merit of the device. The classical detection theory for bulk materials usually divides the device effective area into the optical effective area *A*_d_ and the electrical effective area *A*_e_^5^. Among them, several kinds of electrical effective areas are defined as phenomenological parameters mainly used to estimate various types of electrical noise. For example, when estimating the thermal generation and recombination noise of the device, the total area of the device is usually taken as *A*_e_^5^. For another example, to estimate the Johnson noise of the device, it is necessary to define an effective junction area as the *A*_e_. There are various types of materials and structures for 2D devices, and the contemporary understanding of physical effects limits the effective application of noise models to these devices. Although very convenient, the estimation of *A*_e_ can be inaccurate and, if conditions permit, should be replaced by the measurement of the noise spectrum to derive related figures of merit.

The optical effective area *A*_d_, which are often denoted as *A*_o_ in other works, plays two major roles in characterizing the photodetector: the incident light power is obtained by multiplying *A*_d_ with the signal light power density; the shot noise power corresponding to the signal light and the background radiation is also proportional to *A*_d_^5,6^. The shot noise of the background radiation defines the theoretical upper limit of the specific detectivity of the photodetector in the corresponding application scenario. However, if the noise of the device in the background radiation is measured directly, the only application of the *A*_d_ is to derive the signal optical power.

**Fig. 1:**
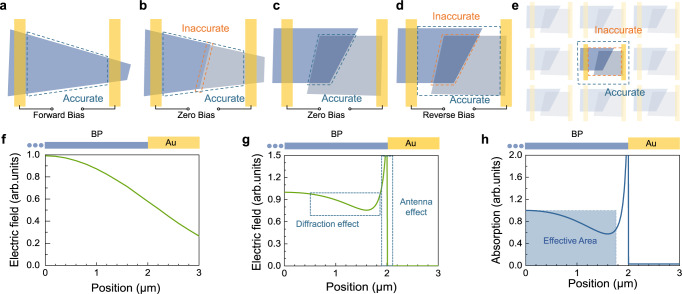
Effective area of different types of 2D photodetectors. **a** Photoconductive photodetector. **b** Planar junction photodetector. **c**, **d** Vertical junction photodetectors with zero and reverse bias, respectively. **e** Focal plane photodetector. The dashed blue lines in **a**–**e** are suggested accurate effective areas. The dashed orange lines in **b**, **d**, and **e** are potential inaccurate effective areas for respective types. **f** Field intensity of the Gaussian beam with the beam waist *w*_0_ = 2.66 μm, here BP represents black phosphorus. **g** Wave optics simulation result of the electric field distribution at the upper surface of the device with plane wave injected. **h** Calculated absorption with the Gaussian beam with the beam waist *w*_0_ = 2.66 μm multiplying the wave optics simulation profile shown in (**g**).

**Fig. 2:**
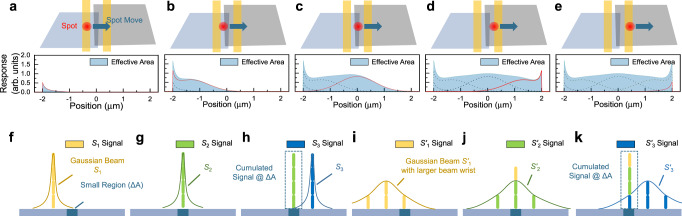
Characterizing the effective area by the scanning photocurrent mapping. **a**–**e** Photocurrent mapping illustration of the distribution of the response with beam centered at different positions with optical responses shown in Fig. 1g. **f**–**k** Illustration of cumulated mapping signal with different beam wrist. *S*_1_, *S*_2_, and *S*_3_ in **f**–**h** are scanning photocurrent signals of Gaussian beam with beam wrist comparable to the length of the small region (denoted as $\Delta A$) near the characteristic length at different positions. *S’*_1_, *S’*_2_, and *S’*_3_ in **i**–**k** are the signals with beam wrist larger than the characteristic length. The dashed blue lines in **h** and **k** show the cumulated signal at $\Delta A$, which are equal in the two cases.

Due to the abundance of 2D device types, working conditions and measurement methods, although *A*_*e*_ is recommended to be discarded, the definition of optical effective area in 2D devices also needs to be classified, including but not limited to the following cases (Fig. 1): (i) For photoconductive devices, the photogenerated electron-hole pairs generated in the region between the two electrodes can all contribute to the photoconductive effect, so *A*_d_ should be taken as all the area covered by the photoelectric materials between electrodes; (ii) For in-plane junction devices operating under zero-bias or reverse-bias conditions, since the width of the junction is difficult to estimate accurately, in order not to cause vast errors, it is recommended to define *A*_d_ as all the area covered by the photoelectric materials between electrodes; (iii) For the vertical junction device working under zero-bias condition, the photocurrent mapping results in most works show that the photoelectric response is mainly relying on the junction area. Without loss of generality, the area of the junction area can be regarded as *A*_d_ in this case; (iv) Once the vertical junction works under the reverse-bias condition, due to the potential drop outside the junction, it is no longer accurate to take the junction area as its optically effective area, and it is necessary to take the area of all materials between electrodes as its *A*_d_; (v) For plane array devices, the entire device tiling the plane needs to be regarded as the *A*_d_ to calculate the responsivity; (vi) For the case where the focused spot irradiates a device with a particularly small channel width, it is necessary to consider both the nonuniformity of the power distribution the spot and the near-field optical effect of the device. Moreover, as is shown in Fig. 2a–e, owing to the high degree of freedom in fabricating the 2D device, a larger *A*_d_ can also be achieved intentionally by electrode vertexes and edges, antennas, and other enhancement microstructures. To effectively characterize the effective area of these types of devices and analyse the influence of background radiation noise on the device, it is recommended to use the photocurrent mapping method, ${A}_{o}\left(\lambda \right)=\int {I}_{s}\left(x,y,\lambda \right){dxdy}/{{\max }}\left[{I}_{s}\left(x,y,\lambda \right)\right]$, where ${I}_{s}\left(x,y,\lambda \right)$ is the photocurrent mapping result of the focused spot centered at the position $\left(x,y\right)$. As shown in Fig. 2a–e, the mapping photocurrent is the weighted average of the intensity of the photocurrent excited by the spot at different positions of the device. The size of the light spot may be larger than the characteristic length of effects influencing *A*_d_ mentioned above, especially in the infrared. The photoresponse of the light spot at each position can also be added up by finely sweeping the spot over the device area, as is shown in Fig. 2f–k, and the antenna effect and diffraction effect can be more accurately included. Moreover, the influence of the spot size on the effective area is weakened.

In characterizations, it is difficult to completely avoid the inhomogeneity of the Gaussian spot of the laser source^7^. By increasing the beam waist radius, the spot can be approximately taken as uniform at the device scale. The intensity distribution of a Gaussian beam at the beam waist has the form $I\left(r\right)\propto {{\exp }}(-2{r}^{2}/{w}_{0}^{2})$, where $r=\sqrt{{x}^{2}+{y}^{2}}$ and ${w}_{0}$ are the radial distance and the beam waist radius, respectively. Assuming that the radius of the circumcircle of the device outline is ${r}_{d}$, and the center of the Gaussian beam is at the center of the circumcircle of the device outline, we have an upper bound estimation of the relative error $\delta$ led by the inhomogeneity of the light spot intensity as $\delta < {\delta }_{U}=\left(I\left(0\right)-I\left({r}_{d}\right)\right)/I\left({r}_{d}\right)={{\exp }}\left(2{r}_{d}^{2}/{w}_{0}^{2}\right)-1$. When the waist radius ${w}_{0}$ is much larger than the radius ${r}_{d}$, we further have $\delta < {\delta }_{U}\approx 2{r}_{d}^{2}/{w}_{0}^{2}$. A simple calculation shows that the upper bound estimation of the relative error is smaller than 1% when ${r}_{d} < \frac{{w}_{0}}{14.18}$, which can be taken as a uniform field in many applications.

## Responsivity

**Fig. 3:**
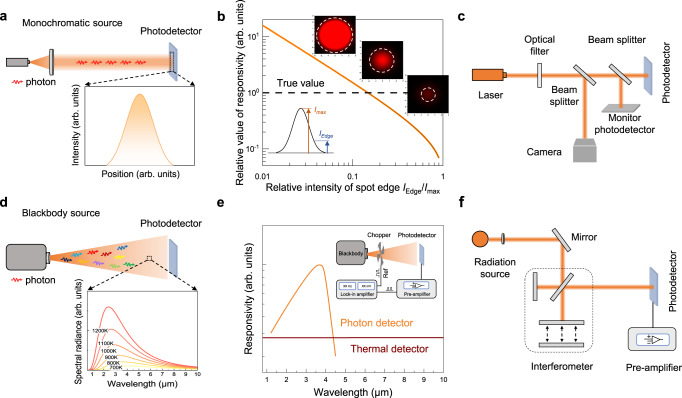
Responsivity and response spectrum determination of 2D photodetectors. **a** Monochromatic laser source measurement system, where the laser spot intensity follows the Gaussian distribution. **b** Relative intensity of the edge of the spot under the researcher’s estimation. The inset shows three spots with the same beam waist and color limit, the only difference of which is the beam intensity. with different intensities and the same beam waist. The estimated radius of spot size shows vast differences. **c** Laser spot size and power calibration measurement system. **d** Photon composition of blackbody radiation source, and the radiation distribution in accordance with Planck’s law. **e** Typical response spectrum of photon detector and thermal detector. The inset shows a diagram of the blackbody measurement system. **f** Schematic diagram of FTIR measurement system.

The responsivity (*R*) is defined as the ratio of the photocurrent or photovoltage of the photodetector to the incident light power^8^. At present, the photocurrent or photovoltage characteristics of most 2D photodetectors are measured by laser, and 2D photodetectors with blackbody response are rarely reported. The laser has better monochromaticity, but the light power intensity is not uniformly distributed (Fig. 3a)^9^. There are many measurement errors and complicated operation problems in the manual calibration of laser power intensity. It should be noted that the use of diffuse laser spot and the spot diameter obtained by human eye observation, and then the normalized power density estimation method will introduce serious errors. On the one hand, due to psychophysical limitations, the estimation of power by human eyes is affected by many factors (environmental brightness, light intensity change gradient, light wavelength, etc.), so it is difficult to effectively estimate the spot diameter. To quantitively model the effect of different researcher’s estimation, a ratio ${I}_{{Edge}}/{I}_{\max }$ is introduced. For a Gaussian beam, the radius of spot $r$ could be derived by ${I}_{{Edge}}/{I}_{\max }={{\exp }}(-2{r}^{2}/{w}_{0}^{2})$, as well as the area $\pi {r}^{2}$ and the power density $P/\pi {r}^{2}$, where $P$ is the power of the spot. For different value of ${I}_{{Edge}}/{I}_{\max }$. The calculation of the relative value of responsivity cancels the beam wrist ${w}_{0}^{2}$ out and are plotted in Fig. 3b. On the other hand, when using the analytic model for spot diameter estimation, the errors generated in the estimation of the parameters of the light source and the piston or tilt error of the optical system will also decrease the accuracy of the estimated power density. These sources of uncertainty can hardly be eliminated by using more complex models of the optical system. For the incident light power density of monochromatic light, it is suggested to calibrate with a standard commercial photodetector (Fig. 3c). The incident light power density at a specific spatial location can be obtained and calibrated by measuring the response of standard commercial photodetectors with defined device effective area under different output powers. With different output signals, the units of responsivity mainly focus on V/W and A/W, where the units could be converted through a transimpedance Z by $R(V/W)=Z\,R(A/W)$^8,10^. In addition, quantum efficiency is another important parameter proportional to the responsivity and is divided into external quantum efficiency (EQE) and internal quantum efficiency (IQE). EQE is defined as the ratio of the number of the electrons collected by the contact to the number of injecting photons. IQE is derived from EQE by dividing absorption (1-Reflection-Transmission).

As a measurement standard of practical application, it is suggested to use a blackbody radiation source to characterize the infrared detection performance of 2D photodetector. Figure 3e shows the schematic diagram of the blackbody measurement system. The total incident power on the photodetector using a blackbody can be expressed as $P={A}_{d}\cdot \frac{\alpha \varepsilon \sigma \left({T}_{b}^{4}-{T}_{0}^{4}\right){A}_{b}}{\pi {L}^{2}}$, where *A*_*d*_ is the device effective area of the photodetector, *α* is the modulation factor, $\sigma$ is the Stefan-Boltzmann constant, $\varepsilon$ is the effective emissivity of the blackbody, *T*_*b*_ is the blackbody temperature, *T*_0_ is the background temperature, *A*_*b*_ is the aperture area of the blackbody, and *L* is the length between the blackbody aperture and the photodetector. The radiation signal (blackbody response photovoltage or photocurrent) is obtained with a chopper and lock-in amplifier. Finally, a blackbody responsivity of the photodetector is acquired. The responsivity can be further divided into spectral responsivity and blackbody responsivity according to different radiation sources. The responsivity spectrum of the photodetector can be obtained by a Fourier transform infrared spectroscopy (FTIR) measurement system and a blackbody measurement system. The response of photon detector varies with the wavelength of incident light, which can be explained by photoelectric effect. The response of the thermal detector depends on the absorbed radiation power, which is independent of the wavelength, and its spectral response curve is a flat line.

## Dark current and noise

Dark current is an intrinsic characteristic parameter of the photodetector, which is obtained when the photodetector is not subjected to external optical radiation. Standardization dark current-voltage (dark *I–V*) measurements should be carried out with cold shield to suppress the background radiation. Especially for 2D infrared photodetectors with narrow bandgap of less than 1.2 eV, the dark current is susceptible to background radiation interference. As a result, compared with *I–V* characteristic, *R*_*d*_*–V* (${R}_{d}={dV}/{dI}$) is more suitable for analyzing the dominant component of dark current in infrared band, which eliminates the interference of background radiation.

**Fig. 4:**
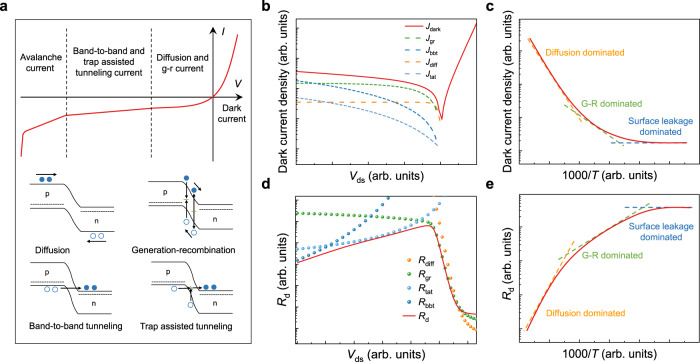
Characterization and analysis of dark current of 2D photodetectors. **a** Typical dark current mechanism, the dashed lines, filled and empty circles and arrows represent quasi-fermi level, electrons, holes, and carrier transport direction. **b** Characterization and analysis of dark current for UV-VIS photodetectors. The solid red line is the *I*_*d*_*–V* characteristic curve measured with a typical VIS photodetector. The green, dark blue, orange, and light blue dashed lines represent the fitted current components of generation-recombination, band-to-band tunneling, diffusion, and trap-assisted tunneling with analytic model. **c** Dominant dark current for typical photovoltaic photodetectors at different temperatures. **d** Characterization and analysis of dynamic resistance for infrared photodetectors. The solid red line is the *R*_*d*_–*V* characteristic curve measured with a typical infrared photodetector. The orange, green, light blue, and dark blue dashed lines represent the fitted current components of diffusion, generation-recombination, trap-assisted tunneling, and band-to-band tunneling with analytic model. **e** Dynamic resistance of typical photovoltaic photodetectors at different temperatures.

Figure 4a summarizes four typical dark current mechanisms. As the reverse bias voltage of the photodetector based on the junction increases, the diffusion current saturates first, which reflects the dark current characteristics of the diffusion limit (including the g-r current). As the reverse bias continues to increase, the tunneling current begins to dominate, followed by the impact ionization current. The analysis of electrical characteristics with technology computer aided design (TCAD) simulation is commonly used to extract different dark current components. Figure 4b, c presents the fitting analysis diagram of the dark current components for 2D ultraviolet-visible (UV-VIS) photodetector and the dominant dark current at different temperatures. Figure 4d, e shows the fitting analysis results of the dynamic resistance of 2D infrared photodetector as a function of bias voltage and temperature.

The noise of 2D materials is difficult to be estimated directly from the dark current due to complex noise components^11^. Moreover, noise- and noise-related detectivity are frequency-dependent quantities. As a result, there is no practical point in discussing noise and noise-related detectivity when the frequency and response bandwidth of the application scenario are ignored. Table 2 summarizes several dominant electric noise mechanisms that are of greatest concern in photodetectors. (i) Thermal noise is the current fluctuation deviating from the average value caused by the irregular thermal motion of the carriers in the resistance. It should not be neglected in 2D photodetectors operating at room temperature. (ii) Shot noise originates from the discrete nature of carriers, which can be described by a Poisson process. It is the most commonly considered noise mechanism in 2D photodetectors. (iii) G-r noise originates from the random fluctuations of emission and capture of carriers from the generation recombination center and trap center at deep levels near the middle of the bandgap. It is ubiquitous and even dominant in 2D photodetectors with photoconductive gain. Ignoring this noise component in photoconductive photodetectors may result in erroneous performance evaluation. (iv) 1/*f* noise (flicker noise) describes the random fluctuation phenomenon of power spectral density inversely proportional to the frequency. This low-frequency noise with significant value reflects the internal quality and reliability of the device. However, the source of 1/*f* noise is still controversial. It should be noted that the thermal noise and shot noise dominate only at high frequencies.

**Table 2:** Dominant noise mechanisms of 2D photodetectors

Noise mechanism	Thermal noise	Shot noise	g-r noise	1/*f* noise
	$\left\langle {i}_{{th}}^{2}\right\rangle=\frac{4{K}_{B}T}{{R}_{d}}\triangle f$	$\left\langle {i}_{{sh}}^{2}\right\rangle=2{qI}\triangle f$	$\left\langle {i}_{{gr}}^{2}\right\rangle=\frac{4{\overline{{\varDelta {N}^{2}}}}\tau }{1+{4\pi }^{2}{{f}^{2}\tau }^{2}}$	$\left\langle {i}_{1/f}^{2}\right\rangle=\frac{{{{\alpha }_{H}I}_{S}}^{2}}{{fN}}\triangle f$
Noise characteristics	White noise characteristic dominated at high frequency		For 2D photodetectors of photoconductive gain with a prolonged carrier lifetime $\tau$ from photogenerated carriers trapped by impurities and defects, g-r noise is proportional to photoconductive gain $\langle {i}_{{gr}}^{2}\rangle=4qI_{ph}G\triangle f$ when ${4\pi }^{2}{{f}^{2}\tau }^{2}\ll 1$	For 2D photodetectors with complex defect state, 1/*f* noise related to random fluctuation of carrier concentration and mobility should not be neglected
	It should not be neglected in 2D photodetectors operating at room temperature	It has usually been considered as the only noise component by the early published work on 2D photodetectors		

*k*_*B*_ is the Boltzmann constant, *T* is the measurement temperature, ${R}_{d}$ is the resistance, and $\,\triangle f$ is the operating bandwidth of the photodetector. $q$ is the electron charge, ${I}$ is the mean current intensity. $\bar{\triangle {N}^{2}}$ represents the mean square fluctuation of the number of carriers occupying the generation-recombination energy level. $\tau={\left(1/{\tau }_{1}+1/{\tau }_{0}\right)}^{-1}$ is the characteristic time constant related to the temperature, where the lifetime $\,{\tau }_{1}$ and ${\tau }_{0}$ is the average duration of the electron in the conduction band and trap level, respectively. ${\alpha }_{H}$ is the Hooge experiential parameter, *N* is the number of carriers, and ${I}_{s}$ is the net photocurrent. $I_{ph}$ is the average photocurrent, $G={\tau }/{{\tau }_{t}}$ is the photoconductive gain.

For 2D photodetectors of photoconductive gain with a prolonged carrier lifetime from photogenerated carriers trapped by impurities and defects, the g-r noise is proportional to photoconductive gain $\langle {i}_{gr}^{2}\rangle=4qIG\Delta {f}$ when ${4\pi }^{2}{{f}^{2}\tau }^{2}\ll \,1$. Without considering 1/*f* noise, g-r noise is the dominant noise for photoconductive photodetector. For photovoltaic photodetectors, the dominant noise mechanism is the shot noise of the current flowing through the pn junction. The total current intensity in pn junction is composed of forward and reverse current components $\left(\right.\langle {i}_{{sh}}^{2}\rangle=2{qI}\triangle f=2q({I}_{0}{e}^{{qV}/{k}_{B}T}+{I}_{0})\triangle f$, where ${I}_{0}{e}^{{qV}/{k}_{B}T}$ is the forward diffusion current, ${I}_{0}$ is the reverse current). At zero bias (*V* = 0), $\langle {i}_{{sh}}^{2}\rangle=4q{I}_{0}\triangle f=\frac{4{k}_{B}T}{{R}_{d}}\triangle f$. As a result, the shot noise at zero bias for photovoltaic photodetectors is also called thermal noise. In addition, the complex defect state and interface effect is unfavorable to 1/*f* noise in 2D photodetectors. So, it is inadequate to ignore 1/*f* noise without any experimental evidence.

**Fig. 5:**
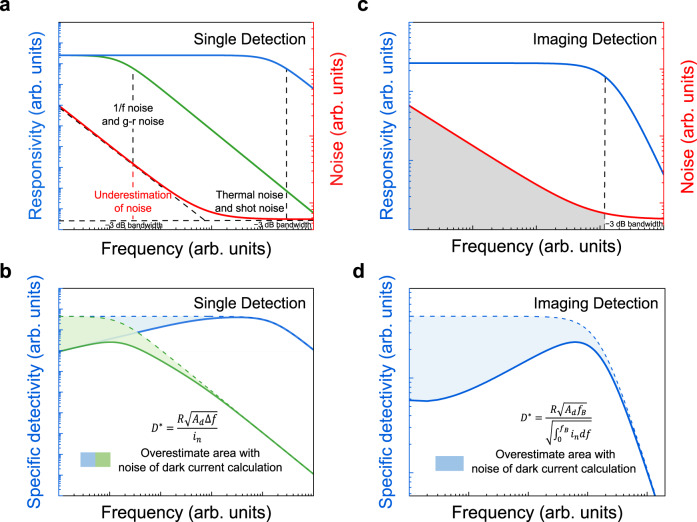
Impact of different noise conditions on D* in scenarios of single detection and imaging detection. **a** Noise and responsivity characteristics for photodetectors with different response bandwidths for single detection (the blue line represents the typical responsivity curve of photodetectors of high response bandwidth, the green line represents the typical responsivity curve of photodetectors of low response bandwidth, and the red line represents the typical noise characteristics. The vertical dashed lines represent the −3 dB bandwidth for photodetectors with high and low response bandwidth). **b** Overestimation of specific detectivity based on noise characteristics for single detection. The solid and dashed lines present the calculated specific detectivity with ${D}^{*}=\frac{R\sqrt{{A}_{d}\Delta f}}{{i}_{n}}$ from the measured noise and estimated noise of thermal noise and shot noise (ignoring the 1/*f* noise and g-r noise). **c** Noise and responsivity characteristics for photodetectors of imaging detection. **d** Overestimation of specific detectivity based on noise characteristics for imaging detection. The solid and dashed lines present the calculated specific detectivity with ${D}^{*}=\frac{R\sqrt{{A}_{d}{f}_{B}}}{\sqrt{{\int }_{0}^{{f}_{B}}{i}_{n}{df}}}$ from the measured noise and estimated noise of thermal noise and shot noise (ignoring the 1/*f* noise and g-r noise).

Figure 5 shows the impact of different noise conditions on D* in scenarios of single detection and imaging detection. Figure 5a, b presents the overestimation of D* for 2D photodetectors with different response bandwidths. Only when the photodetector responds at high frequency, the device noise can be estimated with the white noise of thermal noise and shot noise (${I}_{n}=\sqrt{2{qI}\triangle f+\frac{4{k}_{B}T}{{R}_{d}}\triangle f}$). Especially for photodetectors of high photogating gain, whose response bandwidth is limited by the gain-bandwidth product, the g-r noise is proportional to photoconductive gain mixed in low-frequency noise. As a result, ignoring 1/*f* noise and g-r noise will substantially result in overestimated D* beyond background-limited performance (BLIP). Figure 5c, d presents the responsivity, noise, and D* in the application of focal plane imaging. Here, the fluctuation of signal amplitude caused by noise between different pixels and the same pixel in different frames will both lead to the degradation of imaging quality. The most commonly used focal plane array imaging pattern is to read out the signal after a period of integration, which is equivalent to a low-pass filter for noise. The signal integration process will filter out high-frequency noise. Therefore, when calculating device noise, only the noise at and below the operating frequency needs to be integrated. When testing focal plane devices for engineering applications, the fluctuations of signals of all the pixels in different frames are usually directly regarded as noise. Ignoring the influence of the window function, the method integrating all the noise at and below the operating frequency is numerically close to the method of calculating the signal fluctuations of different pixels.

## Noise equivalent power and specific detectivity

Noise equivalent power (NEP) is defined as the signal optical power incident on the photodetector when the detection signal-to-noise ratio is 1. NEP characterizes the smallest optical signal power that can be resolved from photodetector noise. To obtain an accurate NEP, it is necessary to ensure that the operating frequency of the device is lower than that of the bandwidth of the measurement setups, which is limited by both Nyquist sampling frequency of the noise analysis system and frequency filtering components of measurement setups.

The NEP of most photodetectors is related to the effective area *A*_*d*_ of the photodetector and the electronic bandwidth *f*_*c*_ of the measurement system. It is difficult to compare the performance of different photodetectors with NEP, therefore the D* is introduced to avoid the influence of different effective areas or measurement electronic bandwidth. D* represents the signal-to-noise ratio generated by each unit of irradiation power of the photodetector under unit bandwidth and unit area. Determining the effective area of the 2D photodetector is critical for D* calculation, which has been discussed in detail in previous sections. Besides, the frequency for calculating D* needs to be clearly stated, and the response bandwidth and noise bandwidth during measurement should be consistent^12^.

## Time parameters

**Fig. 6:**
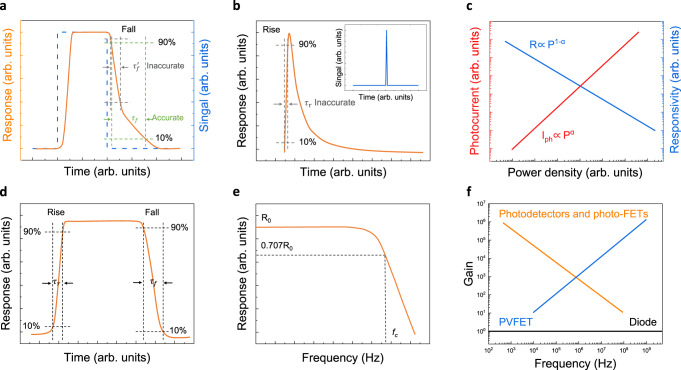
Time parameters and gain determination of 2D photodetectors. **a** Calculated fall time does not reach a stable value which is inaccurate, where ${\tau }_{f}^{{\prime} }$ is inaccurate calculated fall time, ${\tau }_{f}$ is accurate calculated fall time. (The bule line represents the square signal curve, the yellow line represents the typical response curve of 2D photodetectors.) **b** Response time measurement of photodetector may not reach a stable value under pulse signal, which will lead to an inaccurate result. The inset shows pulse signal. The ${\tau }_{r}$ is inaccurate calculated rise time. **c** Variation of photocurrent and responsivity of photoconductive photodetectors with the incident optical power density^14^. **d** Rise and fall response time of photodetector should be calculated from a complete p**e**riodic signal. **e** Typical −3 dB bandwidth response curve of photodetector, where ${R}_{0}$ represents stable responsivity value, ${f}_{c}$ represents the −3 dB cutoff frequency. **f** Gain-bandwidth product of various photodetectors, where photo-FET is photo-field-effect transistor, PVFET is photovoltage field-effect transistor^14^.

For the time parameter of 2D photodetectors, the time required for the output current of the photodetector to rise to a stable response value or to fall to the response value before irradiation is the response time (*τ*). The rise time (*τ*_*r*_) is the time taken for the photocurrent to increase from 10% to 90%, and the fall time (*τ*_*f*_) is the time taken for the photocurrent to decrease from 90% to 10% (Fig. 6d). When measuring the response time of the photodetector, the rise time and fall time are not calculated from a complete response cycle signal, or the output response signal does not rise or fall to a stable value (Fig. 6a, b). The response signal of the photodetector may be not the stable response value, which is not a standardized measurement and can lead to an underestimation of the result.

Alternatively, the response time of the photodetector can be derived from the response bandwidth of the photodetector. For most photodetectors, the responsivity decays as the frequency of the incident light increases. The responsivity of a typical photoconductive photodetector obeys the equation $R\left(f\right)=\frac{{R}_{0}}{\sqrt{1+4{\pi }^{2}{f}^{2}{\tau }^{2}}}$, where ${R}_{0}$ is the responsivity measured under constant irradiation, and the frequency at which the responsivity is reduced to 0.707 times of ${R}_{0}$ is defined as the cutoff frequency *f*_*c*_ (Fig. 6e). The response bandwidth *f*_*c*_ is also known as −3 dB bandwidth.

The gain in a photoconductive detector is proportional to the carrier lifetime whereas the response bandwidth is inversely proportional to the carrier lifetime (Fig. 6c)^13^. Therefore, the gain-bandwidth product of the photodetector dominated by the photoconductive effect is limited (Fig. 6f). Generally, phototransistors with gain dominance can comprehensively consider the following two processes: the generation to recombination process of photogenerated carriers and the channel conductance regulation process of the potential generated by the photocarrier distribution^14^. The final response bandwidth of the photodetector ultimately depends on the smaller bandwidth of the two processes.

## Summary

In this review, we focused on how to characterize figures of merit of 2D photodetectors. Through mechanism analysis and performance evaluation, it is found that the specific detectivity of 2D photodetectors is often overestimated, which is mainly caused by improper calculation of noise, misestimation of device active area and incident light power density. In addition, inconsistent bandwidth of measured responsivity and noise is another primary reason for overestimation of detectivity. The best practices for the measurement of response times and response bandwidths of 2D photodetectors are discussed. Our proposal provides practical guidelines for the standardized characterization of 2D photodetectors, favouring the comparison of the performance of different devices. Meanwhile, this proposal will help promoting the rapid development of 2D photodetectors in industrial areas.
